# Automated cardiovascular risk categorization through AI-driven coronary calcium quantification in cardiac PET acquired attenuation correction CT

**DOI:** 10.1007/s12350-022-03047-9

**Published:** 2022-07-18

**Authors:** S. G. M. van Velzen, M. M. Dobrolinska, P. Knaapen, R. L. M. van Herten, R. Jukema, I. Danad, R. H. J. A. Slart, M. J. W. Greuter, I. Išgum

**Affiliations:** 1 Department of Biomedical Engineering and Physics, Amsterdam UMC location University of Amsterdam, Meibergdreef 123, 1105 AZ Amsterdam, the Netherlands; 2 Informatics Institute, University of Amsterdam, Amsterdam, the Netherlands; 3 Amsterdam Cardiovascular Sciences, Heart Failure & Arrhythmias, Amsterdam, the Netherlands; 4 Medical Imaging Center, Departments of Radiology, Nuclear Medicine and Molecular Imaging, University of Groningen, University Medical Center Groningen, PO Box 30.001, 9700 RB Groningen, the Netherlands; 5 Department of Cardiology, VU University Medical Center, Amsterdam, the Netherlands; 6 Department of Biomedical Photonic Imaging, Faculty of Science and Technology, University of Twente, Drienerlolaan 5, 7522 NB Enschede, the Netherlands; 7 Department of Robotics and Mechatronics, Faculty of Electrical Engineering, Mathematics & Computer Science, University of Twente, P.O. Box 217, 7500 AE Enschede, the Netherlands; 8 Department of Radiology and Nuclear Medicine, Amsterdam UMC location University of Amsterdam, Amsterdam, the Netherlands

**Keywords:** CAD, atherosclerosis, CT, image analysis, artificial intelligence, risk categorization

## Abstract

**Background:**

We present an automatic method for coronary artery calcium (CAC) quantification and cardiovascular risk categorization in CT attenuation correction (CTAC) scans acquired at rest and stress during cardiac PET/CT. The method segments CAC according to visual assessment rather than the commonly used CT-number threshold.

**Methods:**

The method decomposes an image containing CAC into a synthetic image without CAC and an image showing only CAC. Extensive evaluation was performed in a set of 98 patients, each having rest and stress CTAC scans and a dedicated calcium scoring CT (CSCT). Standard manual calcium scoring in CSCT provided the reference standard.

**Results:**

The interscan reproducibility of CAC quantification computed as average absolute relative differences between CTAC and CSCT scan pairs was 75% and 85% at rest and stress using the automatic method compared to 121% and 114% using clinical calcium scoring. Agreement between automatic risk assessment in CTAC and clinical risk categorization in CSCT resulted in linearly weighted kappa of 0.65 compared to 0.40 between CTAC and CSCT using clinically used calcium scoring.

**Conclusion:**

The increased interscan reproducibility achieved by our method may allow routine cardiovascular risk assessment in CTAC, potentially relieving the need for dedicated CSCT.

**Supplementary Information:**

The online version contains supplementary material available at 10.1007/s12350-022-03047-9.

## Introduction

Coronary artery calcium (CAC) is a marker of atherosclerosis^1^ and its presence and the amount increase the risk of any cardiovascular event in symptomatic and asymptomatic individuals.^2-4^ Moreover, the amount of CAC is associated with the likelihood of myocardial ischemia.^5^ Traditionally, CAC is scored manually by an expert in dedicated, ECG-triggered, non-contrast, calcium scoring CT (CSCT) scans, following a previously described and strictly defined method.^6^ However, CAC can be quantified in any CT scan visualizing the heart,^7^ including CT attenuation correction scans that are acquired in nuclear imaging of the heart.^8-10^ As recommended by the Society of Cardiovascular Computed Tomography and Society of Thoracic Radiology (SCCT/STR), CAC derived from CTAC scans enables risk categorization and should be reported, although there is still insufficient evidence on which method to use.^11^

Specifically, in clinical practice, during the myocardial perfusion PET and SPECT scan, a CT attenuation correction (CTAC) scan is acquired that visualizes the heart. If the CTAC could be used for calcium scoring, the acquisition of CSCT scan could be omitted, which would decrease the radiation dose to the patient and increase time and cost efficiency. However, the acquisition of CTAC images is not optimized for CAC scoring, which hampers visualization of calcium lesions and hence, quantitative CAC measurement from these scans. Furthermore, the clinical CAC scoring procedure, which uses a 130 HU threshold to define calcium lesions and enables their detection, limits the reproducibility of CAC quantification^12^: lesions may remain partly or completely below the threshold, because non-ECG-synchronized CTAC scans are heavily affected by cardiac motion and partial volume effect. Therefore, visual calcium assessment from CTAC, which was previously described by Einstein et al and is known to have good agreement with the reference standard CSCT scans, was a proposed solution.^8,13^ It overcomes the discrepancy in acquisition settings between CTAC and reference standard CSCT scan and enables comparison regardless of vendor, tube voltage, slice thickness, and CT-number threshold. However, visual calcium scoring is inherently subjective and requires valuable expert reading time. Therefore, an automatic method may enable robust and time efficient CAC quantification. Therefore, we optimized and evaluated a fully automatic CAC quantification method that does not rely on the clinically used CT-number threshold^14,15^ in CTAC scans, but instead it is based on visual assessment of CAC. Moreover, we design automated risk categorization based on this automatic CAC quantification. Therefore, the aim of this study is threefold: to present an automatic method for optimized CAC quantification in CTAC scans of myocardial perfusion PET, to present automated risk categorization that reproduces expert visual scoring, and to compare the performance to manual calcium scoring from dedicated CSCT scans.

## Materials and Methods

### Patients and imaging data

In this retrospective study we included 742 consecutive patients (58 ± 10, 53.2% male) who underwent a ^15^*O*-water-PET/CTAC and a dedicated CSCT scan between 2008 and 2014 due to known or suspected coronary artery disease (CAD).^16^ Two CTAC scans per patient were acquired: one in rest and one in stress condition. The latter was induced pharmacologically using intravenous adenosine (140 mg/kg/min) infusion. CTAC scans were acquired on a Gemini TF64 PET/CT scanner (Philips Healthcare, Best, the Netherlands) without contrast enhancement, with 120 kVp and without ECG synchronization. Images were reconstructed to 1.17 mm in-plane resolution and 5 mm slice thickness and increment. Dedicated CSCT scans were acquired on a 256-slice Brilliance iCT-scanner (Philips Healthcare, Best, the Netherlands) without contrast enhancement, with a tube voltage of 120 kVp and with ECG synchronization for optimal visualization of the coronary arteries. Images were reconstructed to 0.36-0.47 mm in-plane resolution and 2.5 mm slice spacing and increment.

In total 50 patients were excluded; 36 were excluded due to incomplete imaging data and 14 were excluded due to uninterpretable images, e.g., severe artifacts due to low dosage (43%) or metal implants (57%), which resulted in a set of 692 patients. The study complied with the Declaration of Helsinki. The need for written informed consent was waived due to the retrospective design of the study by the institutional review board (Medical Ethics Committee of the Amsterdam UMC, Vrije Universiteit Amsterdam).

Additionally, to enhance the training with ample examples of CAC lesions due to a high CAC burden in non-dedicated CT scans, 629 low-dose chest CT acquired at baseline from participants of the National Lung Screening Trial (NLST)^17^ were included. This set was previously used for development of a calcium scoring method and was designed to be diverse with respect to scanner model and reconstruction algorithm.^15,18^ CT scans were, like CTAC, acquired without contrast enhancement and without ECG synchronization. NLST acquisition was made with a tube voltage of 120 kVp or 140 kVp, depending on the subject’s weight. Images were reconstructed to 0.49-0.98 mm in-plane resolution, 1.0-2.5 mm slice thickness, and 0.6-2.5 mm increment. To make the slice thickness uniform in our study, the scans were resampled to 3.0 mm slice thickness and 1.5 mm increment, following earlier studies.^18-20^

For training, a set of all available NLST data and 136 CTAC scans of 68 patients were used. For evaluation two hold-out sets were used: evaluation Set 1 of 100 cardiac patients for comparison with the clinically used method and evaluation Set 2 consisting of 461 cardiac patients for comparison with visual scores only. In both evaluation sets each patient had 2 CTAC scans and a CSCT scan.

### Reference data

The automatic method used in this study consists of several components. To train and evaluate each of these, expert-defined reference data were obtained.

#### Training automatic calcium scoring

Our method aims to quantify calcifications in CTAC scans according to visual assessment rather than using the CT-number threshold for calcium extraction. Nevertheless, manual annotation of calcified voxels in the coronary arteries would be practically infeasible. Therefore, to train the automatic CAC scoring, for each axial CT slice in the training set, the presence of CAC (yes/no) was defined by visual assessment. This was performed by an expert observer (S.G.M.V) with > 5 years of experience in calcium scoring.

Moreover, to limit the analysis to the region of interest only, i.e., to train the automatic heart segmentation in CTAC scans, the heart was manually segmented in a subset of the training data. This set comprised a randomly chosen set of 39 NLST scans and 29 CTAC scans. The segmentation was performed by voxel painting the heart by the same observer (S.G.M.V).

#### Manual calcium scoring

To allow comparison with standard clinically accepted calcium scoring procedure that exploits the intensity-based threshold,^6^ a trained observer (R.L.M.H) manually annotated calcium following the clinical procedure in all CTAC scans and CSCT scans in evaluation Set 1. This was done using in-house custom-built software for semi-automatic calcium scoring,^19,21^ in which the observer identified lesions with ≥ 130 HU. For each scan, CAC was quantified into Agatston score^6^ and assigned to one in five cardiovascular risk categories (I: 0, II: 1-10, III: 11-100, IV: 100-400, V: > 400).^21,22^

#### Visual scores

To allow comparison of the automatic CAC quantification with visual evaluation in CSCT and CTAC, visual scoring was performed in evaluation Set 1 and Set 2 by an expert observer with > 2 years of experience in visual calcium scoring (M.M.D). Visual scoring was performed as described by Einstein et al by estimating the Agatston risk category the scan belonged to.^8^ To enable comparison between clinically used Agatston risk categories, instead of a six-point scale used by Einstein et al, the same five-point scale as described above was used (I: 0, II: 1-10, III: 11-100, IV: 100-400, V: > 400).

### Automating visually assessed CAC quantification

**Figure 1:**
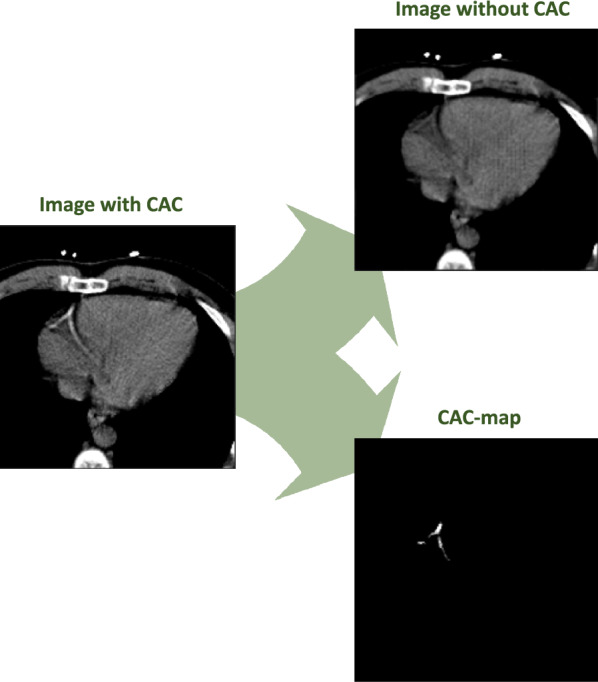
The automatic method decomposes an image with CAC (left) into an image without CAC and a CAC-map

In CTAC scans, CAC scoring is challenged by large slice thickness, low in-plane image resolution, motion artifacts, and image noise. Hence, to detect and quantify CAC lesions partly or completely below the standardly used CT-number threshold, we utilize a method that does not rely on the clinically used CT-number threshold of 130 HU.^14,15^ The method decomposes an image slice containing CAC into its counterpart without CAC and a CAC-map, that is an image indicating area and density of calcifications (Figure 1). In this work, the method is specifically optimized for the CTAC scans. Moreover, to simplify the analysis, we limit generation of CAC-maps to the heart region only.

#### Defining the region of interest

**Figure 2:**
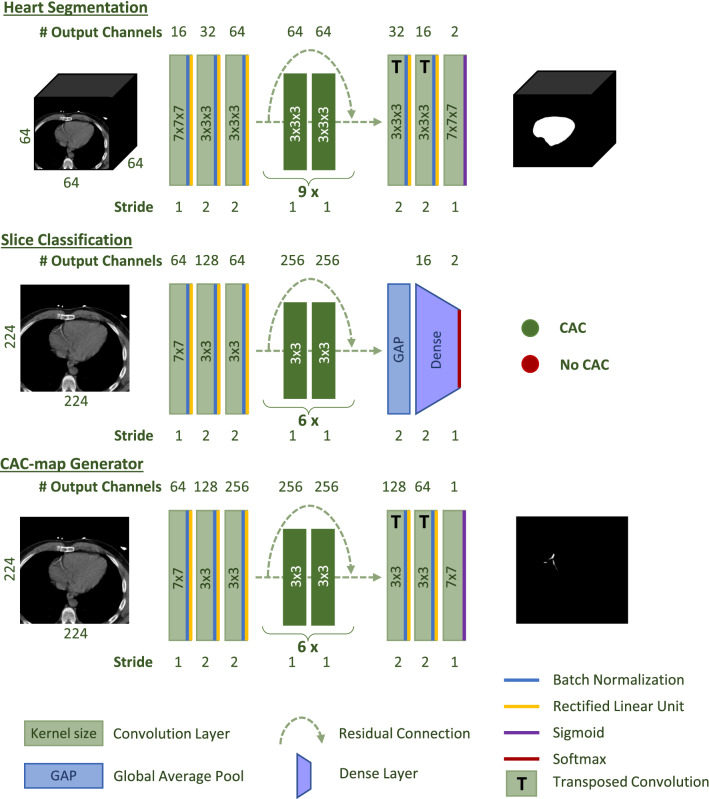
Architecture of the convolutional neural networks used in the automatic CAC quantification method for heart segmentation, slice classification, and generating CAC-maps

Prior to analysis, all images were resampled to 1.0 mm in-plane resolution and 1.5 mm slice spacing. To simplify the task of CAC quantification, we identify the region of interest by heart segmentation. Since manual heart segmentation of a large (training) set is time consuming, the heart is automatically segmented using a 3D convolutional neural network (CNN) with a Resnet architecture (Figure 2). The CNN was trained with a subset of the training set (36 NLST and 26 CTAC scans), with available reference heart segmentations. The trained CNN was used to segment the heart in the remaining training scans and in the evaluation set.

Subsequently, to select slices for CAC quantification during testing, the slices containing CAC were identified through classification according to presence of visible CAC lesions. This was done using a 2D CNN with a Resnet architecture (Figure 2) that was trained using reference labels indicating the presence of CAC in an axial slice.

#### CAC quantification

Manual annotation of CAC by, e.g., mouse painting that is based on visual evaluation, i.e., without using a threshold, is hardly feasible. Hence, a reference standard CAC-map for training a segmentation CNN is practically not attainable. Instead, we use a method that decomposes an image with CAC into an image without CAC and a CAC-map (Figure 1).^14,15^ This is done by generating a synthetic image without CAC from the image containing CAC. The CAC-map is then computed as the difference between the input image with CAC and the synthetic counterpart without CAC. Standardly, generative adversarial networks (GANs) are used for image synthesis, which are often trained using paired images. However, image pairs, i.e., images of the same patient, where one image contains CAC and the other image does not, do not exist. Hence, we use an approach that allows image synthesis using unpaired training: namely, we use a CycleGAN,^23^ a system of CNNs that translates images from one domain to another and vice versa using unpaired training data.

**Figure 3:**
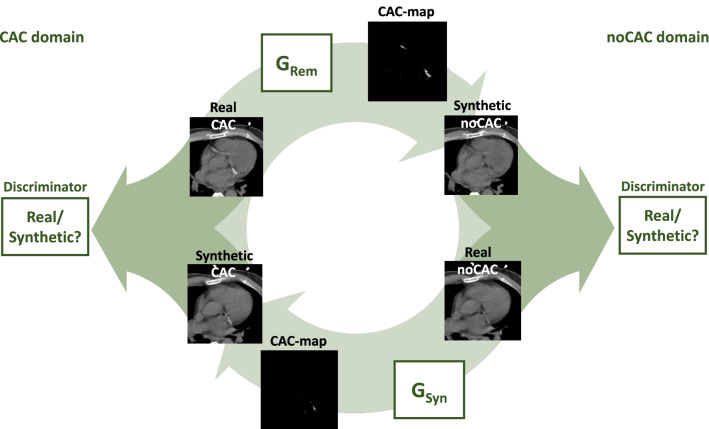
Schematic overview of the CycleGAN used for CAC segmentation, i.e., generating the CAC-maps. Images containing CAC (CAC domain) are translated into images without CAC (noCAC domain) by predicting a CAC-map with the generator (G_Rem_) and subtracting it from the input image. Images from the noCAC domain are translated to the CAC domain by adding the CAC-map, predicted by G_Syn_, to the input image without CAC. The discriminators determine how well the synthesized images match real images in their domain

As proposed in our previous work,^14,15^ one domain is defined by images containing CAC (*CAC domain*) and the other domain by images without CAC (*noCAC domain*) (Figure 3). Hence, the CycleGAN translates *CAC* images into *noCAC* images and vice versa.^14,15^ In this approach, the CAC-map is defined as the difference between the CAC image and the synthetic noCAC counterpart. The CycleGAN contains two generator CNNs that synthesize images: the *removing* generator (G_Rem_) that translates from the *CAC domain* to the *noCAC domain* by generating a CAC-map and subtracting it from the input CAC image and the *synthesizing* generator (G_Syn_) that translates from the *noCAC domain* to the *CAC domain* by generating a CAC-map and adding it to the input noCAC image. Next to these, the CycleGAN contains two discriminator CNNs that determine how well the synthesized images match real images in the target domain. By optimizing the generators and discriminators together, the generators are trained to produce realistic synthetic images and hence, a realistic CAC-map.

To optimize the calcium quantification for the CTAC scans, the CycleGAN is trained using the training set of 629 scans from NLST and 114 CTAC scans (57 rest and 57 stress) of 57 patients that underwent ^15^*O*-water-PET/CTAC imaging. In each training scan the heart was segmented. For training, axial scan slices were assigned to either the CAC or noCAC domain based on the presence of visible CAC in the slice indicated by the reference labels. Given that images with and without CAC of the same patients do not exist, we utilize an unpaired training approach, i.e., CAC and noCAC images originate either from different patients and/or different anatomical levels. The CycleGAN was trained for 375.000 iterations, with a learning rate of 0.0001 using batches of four axial CT slices of 224 × 224 voxels. For more details on, e.g., architecture, loss functions, and hyperparameters for training, we refer to our previous work.^14,15^ After training the CycleGAN, its G_Rem_ was used to obtain the CAC-maps. Thereafter, in CTAC scan, CAC was quantified into CAC pseudomass. To allow comparison with clinical calcium scoring in CSCT, this was done using the original image values of the CAC area as indicated in the CAC-map, instead of directly using the CAC-maps. Since the scans are not calibrated for quantification of CAC mass, we calculate CAC pseudomass, which is the uncalibrated CAC mass. The CAC pseudomass is computed as the sum of the CT-number values in the lesion multiplied by the voxel spacing.^24^

### Automated risk categorization

In clinical care, patients are assigned to a cardiovascular risk category to determine the risk of experiencing a CVD event and the risk of myocardial ischemia.^2,5,25^ The clinically used risk categories are based on Agatston scores that utilize the 130 HU threshold.^6^ Hence, our CAC quantification cannot be directly translated to Agatston scores or risk categories based on the Agatston scores. Moreover, since the CTAC images are heavily affected by cardiac motion artifacts and partial volume effect, previous research shows that visual scoring may have better agreement with Agatston risk categories in CSCT.^8^ Therefore, based on the proposed automatic CAC quantification, the scans were assigned a cardiovascular risk category (${\mathrm{Vis}}_{\mathrm{auto}}$) on a five-point scale that was calibrated using visual scores. For this, the CAC pseudomass is log transformed and calibrated using the following formula:

$$ {\text{Vis}}_{{{\text{auto}}}} = \alpha \cdot \log_{\beta } \left( {\gamma \cdot {\text{CACmass}}} \right) - \delta ;\quad {\text{for}}\;{\text{CACmass}} > 0 $$

$$ {\text{Vis}}_{{{\text{auto}}}} = 0;\quad {\text{for}}\;{\text{CACmass}} = 0 $$

Calibration of the scaling factors ($\alpha$ and $\gamma$), offset ($\delta$), and base of the logarithm ($\beta$) was performed for rest and stress CTAC separately in SPSS 28.^26^ For each scan type, four scans from the training set with a visual score of 2, 3, 4, and 5 were randomly selected, totaling 16 scans. These scans were not used in evaluation in any way. Regression between the CAC pseudomass and visual score of these scans was used to determine $\alpha$, $\beta$, $\gamma$, and $\delta$. The following parameters for calibration were found: $\alpha = 3.1, \beta = 9.0, \gamma = 1.1$ and $\delta =1.6$ for rest CTAC; $\alpha = 3.5, \beta = 9.0, \gamma = 1.0$ and $\delta = 1.5$ for stress CTAC.

### Evaluation

The performance of automatic calcium scoring in CTAC scans was evaluated using standard clinical calcium scoring with a 130 HU threshold in CSCT scans as a reference. For comparison, the amount of CAC in both CTAC and CSCT was quantified with CAC pseudomass. Please note that the scans are not calibrated for CAC mass. Furthermore, automatic scoring in CTAC (without CT-number threshold) was compared with manual clinical calcium scoring following the clinical scoring protocol (using 130 HU threshold).

The performance of the proposed method was evaluated through the interscan reproducibility of CAC quantification, between CTAC and CSCT scan pairs, using average absolute relative differences (${\Delta }_{R}$). This is computed as the absolute difference between CAC scores in CTAC and CSCT divided by their mean:

$$ \Delta_{R} = \frac{{ \left| {{\text{CTAC}} - {\text{CSCT}}} \right|}}{{{1 \mathord{\left/ {\vphantom {1 2}} \right. \kern-\nulldelimiterspace} 2} \left( {{\text{CTAC}} + {\text{CSCT}}} \right)}} $$

Moreover, Bland–Altman plots with 95% limits of agreement were assessed. Because errors tend to increase with increasing amount of CAC, the limits of agreement were determined using regression to model the variation of the absolute differences between measures from CTAC and CSCT and multiplying by $1.96\cdot (\pi /2{)}^{1/2}$ because the absolute differences have a half-normal distribution.^27^

Next, the ability of the automatic method to detect the presence of CAC in CTAC scans using CSCT as the reference was determined using sensitivity, specificity, and F1 score.

Finally, to evaluate whether automated risk categorization in CTAC is feasible, agreement in cardiovascular risk categorization between automatic risk category assignment in CTAC and risk category assignment based on the Agatston scores in CSCT was evaluated using Cohen’s linearly weighted kappa $\left( {\kappa_{LW} } \right)$. Automated risk categorization was compared with both risk categorization based on manual Agatston scores from CTAC and visual scoring performed in CSCT and CTAC, by an observer.

## Results

### Quantification of CAC

Using the described automatic method, CAC was identified in CTAC scans from the evaluation set. The performance was evaluated on the images of patients in the evaluation set in terms of interscan reproducibility of CAC quantification and detection of presence of CAC using standard manual clinical calcium scoring in CSCT as reference.

#### Interscan reproducibility

**Figure 4:**
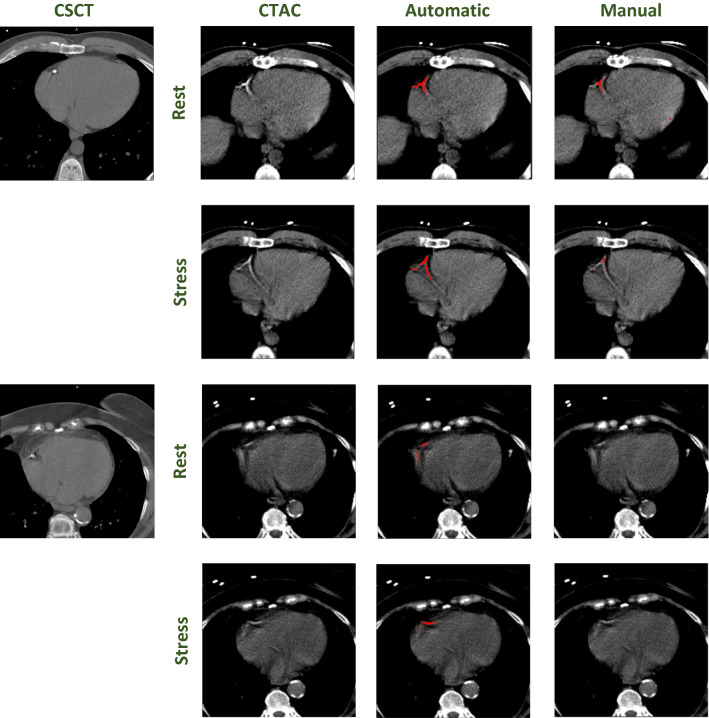
Examples of pairs of CSCT, rest and stress CTAC with CAC lesions. Overlays (red) are shown on the CTAC scans for the automatic method that is independent of the CT-number threshold and manual calcium scoring, which uses the 130 HU threshold

To assess the interscan reproducibility of calcium quantification in CTAC scans in evaluation Set 1, we compared the calcium scores in CTAC using here described automatic (without CT-number threshold) and manual clinical segmentation (with 130 HU threshold) with calcium scores from CSCT obtained using manual clinical procedure (with 130 HU threshold). Using the automatic CAC quantification with pseudomass, the ${\Delta }_{R}$ was 118% for rest scans and 121% for stress scans (Table 1). In concordant scan pairs, i.e., pairs in which CAC was detected in both the CTAC and CSCT scan, ${\Delta }_{R}$ was 75% and 85% in rest and stress scans, respectively. In comparison, with manual clinical calcium scoring a ${\Delta }_{R}$ in CAC pseudomass of 159% in rest scans and 153% in stress scans was found. In concordant pairs the ${\Delta }_{R}$ was 121% and 114% in CAC pseudomass, for rest and stress scans, respectively. For Agatston scores obtained using manual clinical calcium scoring similar results were obtained (Table 1). Figure 4 shows examples of CAC lesions identified on CTAC using the automatic method.

**Table 1:** Average absolute relative difference (${\Delta }_{R}$) between standard manual clinical calcium scoring in CSCT (reference) and automatic CAC quantification or clinical calcium scoring in CTAC

Evaluation set 1 (N = 98)	CTAC vs CSCT	
	Δ_*R*_ (%)	Δ_*R*_ concordant (%)
Automatic CAC quantification (CAC pseudomass)		
Rest CTAC	118	75
Stress CTAC	121	85
Clinical calcium scoring (CAC pseudomass)		
Rest CTAC	159	121
Stress CTAC	153	114
Clinical calcium scoring (Agatston score)		
Rest CTAC	154	135
Stress CTAC	148	126

Results are shown for all pairs in evaluation Set 1 and only concordant pairs in evaluation Set 1, i.e., pairs in which CAC was detected in both the CTAC and CSCT scan

**Figure 5:**
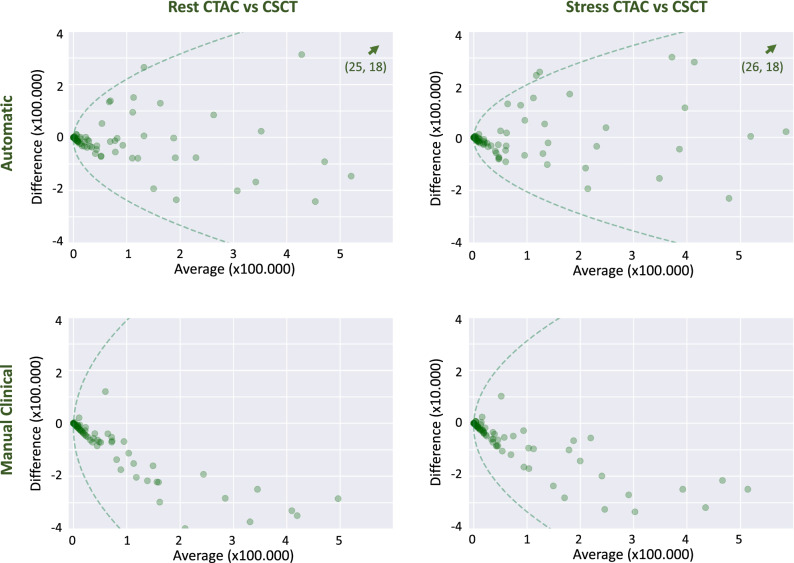
Bland–Altman plots of CAC pseudomass with 95% limits of agreement (dashed lines) comparing manual reference scoring in CSCT with quantification in CTAC using the automatic method (top) and manual calcium scoring (bottom) in evaluation set 1 (N = 98). 95% are represented by the following formula: $\mathrm{Difference }=b \pm 1.96\cdot (\uppi /2{)}^{1/2}\cdot a\cdot {\mathrm{mean}}^{1/2}$. For automatic quantification the coefficients *a* and *b* are 295 and − 8757 in rest CTAC and 261 and − 3210 in stress CTAC. For manual scoring the coefficients *a* and *b* are 524 and − 17,465 in rest CTAC and 416 and − 11,251 in stress CTAC. Outliers are indicated by an arrow

Figure 5 shows Bland–Altman plots with 95% limits of agreement in CAC pseudomass extracted from CTAC scans and CSCT scans in evaluation Set 1. It can be appreciated that the agreement is better for the automatic CAC quantification method than for manual clinical calcium scoring. Moreover, manual clinical calcium scoring shows systematic underestimation (negative differences) of the CAC pseudomass.

#### Detection of presence of CAC

According to manual clinical calcium scoring in CSCT evaluation scans, CAC was present in 67 out of 98 patients (68%) in evaluation Set 1. The automatic method detected CAC in 72% and 77% of these scans (sensitivity) and found 7 and 8 false-positive scans in rest and stress CTAC scans, respectively (Table 2). In comparison, manual clinical calcium scoring in CTAC showed substantially lower sensitivity of 54% and 58%, but with 3 and 4 false-positive scans in rest and stress CTAC scans, respectively. Visual assessment of the false-positive cases of the automatic method revealed that in 53% non-coronary calcium was detected (e.g., in the ascending aorta and cardiac valves), in 13% ring artifacts were detected as CAC, and in 33% image noise in the vicinity of the coronary arteries was detected as CAC.

**Table 2:** Sensitivity, specificity, and F1 score for detection of patients that have a positive score on CSCT according to reference manual calcium scoring in evaluation Set 1

Evaluation set 1 (N = 98)	CTAC vs CSCT		
	Sensitivity	Specificity	F1
Automatic CAC quantification			
Rest CTAC	0.72	0.77	0.79
Stress CTAC	0.76	0.74	0.81
Manual calcium scoring			
Rest CTAC	0.54	0.90	0.68
Stress CTAC	0.58	0.87	0.71

Results are shown for the automatic method and clinical manual calcium scoring in CTAC rest and stress scans

### Automated risk categorization

**Figure 6:**
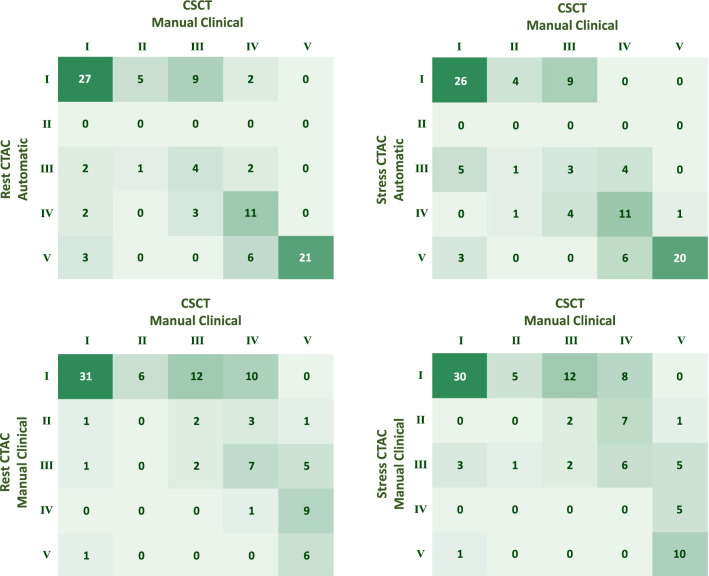
Concordance matrixes of 5-category risk categorization in evaluation set 1 (N = 98) according to manual clinical and automatic calcium quantification in CTAC scans versus manual clinical calcium scoring in CSCT scans

According to visual scoring in CSCT, 67/98 patients (68%) in evaluation Set 1 had CAC. The automated risk categorization in CTAC showed a moderate agreement with expert visual scores in CSCT, with a ${\kappa }_{LW}$ of 0.65 (CI 0.54-0.76) in rest scans and 0.65 (CI 0.55-0.75) in stress scans (Table 3). Similarly, a moderate agreement with the clinical standard, namely with Agatston risk categories determined on CSCT, was found with a ${\kappa }_{LW}$ of 0.65 (CI 0.54-0.77) in rest CTAC and a ${\kappa }_{LW}$ of 0.65 (CI 0.55-0.76) in stress CTAC. Concordance matrixes are shown in Figure 6. When comparing automated risk categorization in CTAC with expert visual scores in the same scans, a good agreement was found with ${\kappa }_{LW}$ of 0.71 (CI 0.60-0.81) in rest scans and 0.73 (CI 0.64-0.83) in stress scans.

**Table 3:** Agreement of risk categorization in CTAC according to the automatic method, manual calcium scoring, and visual scoring

Evaluation set 1 (N = 98)	Reference			
	Manual calcium scoring	Visual scores		
	CSCT	CSCT	Rest CTAC	Stress CTAC
Automated risk categorization				
Rest CTAC	0.65 (0.54–0.77)	0.65 (0.54–0.76)	0.71 (0.60–0.81)	
Stress CTAC	0.65 (0.55–0.76)	0.65 (0.55–0.75)		0.73 (0.64–0.83)
Manual calcium scoring				
Rest CTAC	0.40 (0.29–0.51)			
Stress CTAC	0.40 (0.28–0.52)			
Visual scoring				
Rest CTAC	0.74 (0.65–0.84)	0.76 (0.68–0.84)		
Stress CTAC	0.79 (0.71–0.87)	0.79 (0.71–0.86)		

Risk categorization is compared to manual calcium scoring (Agatston) in CSCT and to visual scores in CSCT and CTAC using linearly weighted kappa with 95% confidence intervals

**Figure 7:**
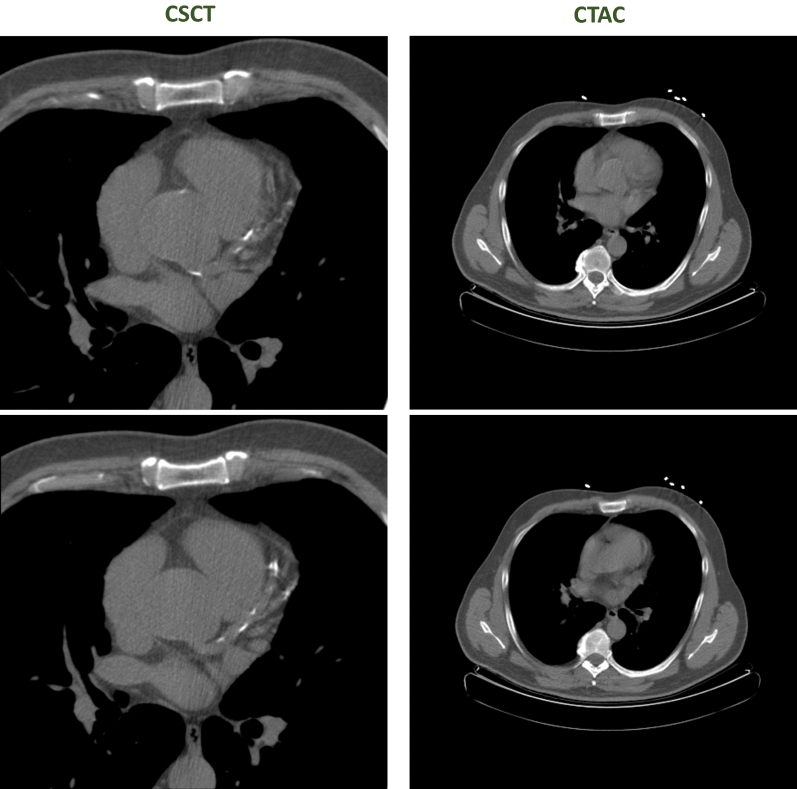
Example of a case where CAC is visible in the CSCT, but not in the rest CTAC of the same patient. Two adjacent slices are shown per scan

The agreement of expert visual scores in CTAC with visual scores in CSCT was lower in evaluation Set 2 with a ${\kappa }_{LW}$ of 0.63 (95% CI 0.58-0.68) in rest CTAC and 0.65 (95% CI 0.60-0.70) in stress CTAC. This was due to false-negative findings, in which CAC was visible in the CSCT but not in the CTAC (Figure 7). In line with this, the agreement between automated risk categorization in CTAC and visual risk categorization in CSCT showed a ${\kappa }_{LW}$ of 0.55 (95% CI 0.55-0.61) in rest CTAC and 0.58 (95% CI 0.52-0.63) in stress CTAC. Comparing automated risk categorization in CTAC with visual scores in CTAC in evaluation Set 2 led to a ${\kappa }_{LW}$ was 0.69 (CI 0.63-0.74) in rest scans and 0.63 (CI 0.58-0.69) in stress scans.

Since a CAC Agatston score > 400 is strongly associated with myocardial ischemia in patients with suspected CAD,^28^ we evaluate detection of high CAC patients from CTAC. Note that manual Agatston scores are only available for evaluation Set 1. Using automated risk categorization 21 and 20 out of 21 patients in evaluation Set 1 with > 400 Agatston score on CSCT were correctly identified in rest and stress CTAC, respectively. In both rest and stress CTAC, 8 out of 77 patients with a score < 400 on the CSCT were incorrectly identified as having a > 400 score by automated risk categorization.

The automatic method correctly classified 5 patients in rest CTAC and 4 patient in stress CTAC out of 22 patients with a reference Agatston score between 1 and 100 on the CSCT. Among these 22 patients, 12 had no visible CAC in the rest CTAC scan and 10 had no visible CAC in the stress CTAC scan. The performance of manual calcium scoring in CTAC was similar, with 4 patients at rest and 5 patients at stress correctly classified. Both the automatic method and manual calcium scoring in CTAC correctly classified zero patients out of 6 with a reference Agatston score between 1 and 10 on the CSCT.

## Discussion

This study presented and evaluated an automatic method for quantification of CAC that is based on visual assessment of CAC and specifically optimized for rest and stress CTAC scans acquired during myocardial perfusion PET/CT scans. CAC quantification using our method enables higher sensitivity of CAC detection and increased interscan reproducibility between CTAC and CSCT of CAC pseudomass compared with clinical calcium scoring in CTAC scans. Moreover, this study presented automated risk categorization from CTAC that showed a higher agreement with Agatston score-based CVD risk categories in CSCT than manual CAC scoring in CTAC following the clinical protocol.

Given that the presented CAC scoring does not depend on a predefined CT-number threshold for manual calcium detection, it addressed the limitations imposed by non-dedicated image acquisition protocols of CTAC scans that hamper manual CAC scoring. Recent trends in CAC scoring from all chest CT scans have led to a proliferation of studies focused on CAC assessment from CTAC scans.^9,10,29^ Mylonas et al achieved high agreement between manual CAC scoring from CTAC and standard clinical CSCT by decreasing the detection threshold to 50 HU.^9^ However, when the conventional 130 HU threshold was applied, the agreement with CSCT scan decreased to *κ* = 0.33. A major drawback of decreasing the detection threshold regardless of scan acquisition parameters is that it does not reflect the Agatston scoring method.^6^ As previously described, the CT-number threshold is dependent on the tube voltage applied in acquisition and should be adjusted accordingly.^30^ Moreover, this CT-number threshold would have to be changed following different PET and SPECT CT scanners and all varieties of protocols with different tube voltage levels. Therefore, our automated risk categorization method enables CAC detection regardless of the used tube voltage.

Moreover, automated CVD risk categorization in CTAC scans strongly outperformed risk categorization based on manual CAC scoring in CTAC scans. This might be explained by a high level of present motion artifacts due to lack of ECG synchronization in the CTAC scans, which cause lesions to (partly) remain below the 130 HU detection threshold used in clinical calcium scoring. Moreover, the slice thickness of CTAC used in this study was 5 mm, instead of 3 mm that is standardly used in calcium scoring, and the in-plane resolution was lower compared to typical CSCT, which both increased the partial volume effect.^31^ Due to partial volume effect, smaller calcifications may remain below the detection threshold and are not recognized as calcium.

The automated risk categorization, which is independent of the 130 HU threshold, gained a higher agreement with standard clinical risk categories from CSCT than manual calcium scoring in CTAC, which does rely on the detection threshold. Nevertheless, an automated method by Išgum et al that utilizes the 130 HU detection threshold reached a higher agreement with CSCT in risk categorization as compared to our automated risk categorization (rest: *κ* = 0.74 vs. *κ* = 0.65, respectively; stress *κ* = 0.70 vs. *κ* = 0.65, respectively).^10^ However, large differences in acquisition parameters (100 kV vs 120 kV; 3 mm vs 5 mm) hamper direct comparison of the results. This is underlined by the difference in agreement of manual calcium scoring in CTAC scans with CSCT: *κ* = 0.85 in the study of Išgum et al vs *κ* = 0.4 in our study. Nevertheless, the here presented method is not limited to the 5 mm CTAC data used in this study, but can also be applied to CT scans with other protocols.^14,15^ Since scans with 3 mm slice thickness allow better visualization of CAC lesions, we expect that the agreement of our method with CSCT scans improves when applied to scans with lower slice thickness.

According to conventional, visual CAC scoring methods, in the study by Einstein et al and Engbers et al 63% and 71% of CAC scores, respectively, were classified to a correct risk category.^8,13^ In our study, 65% of rest CTAC and 62% of stress CTAC scans analyzed with automated risk categorization were assigned to a correct risk category, which shows potential for further improvement. Importantly, as opposed to Einstein et al and Engebers et al, who used a 6-point risk scale,^8,13^ in our study a 5-point risk scale was applied, to facilitate comparison with previous works that performed manual and automatic calcium scoring in CTAC.^9,10^

The agreement between automated risk categories in CTAC and expert visual risk categorization in CSCT was lower in evaluation Set 2 compared to evaluation Set 1. In line with this, the agreement between visual scores in CTAC and visual scores in CSCT was lower than in Set 1. This underlines the challenges presented by the suboptimal acquisition protocol for calcium scoring of the CTAC scans, hampering visualization of lesions and sometimes even making them invisible in the CTAC scans. Future work could evaluate the performance of the method in CTAC scans with thinner slices compared to visual scoring.

As indicated by He et al, the magnitude of CAC and the stress-induced myocardial ischemia determined by PET and SPECT are positively correlated. Moreover, this relation was quantified by dividing the amount of CAC into previously used five risk groups. As a result, almost half of patients with CAC ≥ 400 were also diagnosed with myocardial ischemia.^28^ These findings were further supported in the meta-analysis which revealed that zero and low CAC score is rarely associated with ischemia.^5^ In our study the agreement in risk classification between automated risk categorization and manual reference scoring was moderate (*κ* = 0.65). Nevertheless, all CAC from the rest CTAC were correctly assigned to the ≥ 400 group by automated risk categorization. From stress CTAC scans, 4.7% was underestimated and classified into a lower risk category. This finding indicates that automated risk categorization may improve the likelihood assessment of myocardial ischemia in patients with marginal PET and SPECT results.

A zero CAC score is the most powerful negative predictor of cardiac events in an asymptomatic population of patients.^32^ Moreover, in symptomatic patients, who are referred to myocardial perfusion PET and SPECT scans, a low calcium score indicates a lower risk of CVD events.^2^ This is especially important for patients with normal PET and SPECT results, because the lack of myocardial ischemia does not rule out the presence of atherosclerotic disease. As indicated in our study, the sensitivity and specificity of CAC detection were good which indicates that automated risk categorization based on CTAC is a reliable method for CAC detection. Hence, CAC directly demonstrates the presence of the atherosclerotic process and it may help clinicians to identify patients of a higher cardiovascular risk and initiate a medical therapy, as specified in guidelines.^2-4,11,33-35^ Nevertheless, several patients had a false-negative result, mostly due to CAC lesions that are not visible on the CTAC scan. Moreover, several false-positive detections were found in image noise and ring artifacts. Automatic noise reduction^36^ and artifact removal^37^ may offer a solution.

Our study has several limitations. First, the automatic method is evaluated on a single-center dataset and the CTAC scans were acquired on one PET/CT scanner, therefore a direct comparison of performance with different CTAC protocols was not possible. Second, in this study not a 3 mm slice thickness but 5 mm slice thickness of CTAC scans was applied. Nevertheless, the results demonstrate a good agreement with scoring in CSCT scans which indicates feasibility of the approach. Moreover, we expect further improvement when applied on CTAC acquired with 3 mm slice thickness. Third, both the CSCT and the CTAC scans were not calibrated for CAC mass. However, we expect minor impact on the quantification and expect conclusions to remain the same if corrections would be applied. Fourth, the correspondence between calcifications detected on CTAC scans and CSCT scans was not investigated. Therefore, only patient-based analysis, not lesion-based analysis was performed.

## Conclusion

We presented an automatic method for CAC quantification in CTAC that is based on visual assessment of CAC and independent of the CT-number threshold. The described automatic CAC quantification in CTAC improves the agreement with standard manual calcium scoring in CSCT compared with manual calcium scoring in CSCT using the threshold. Moreover, automatic risk categorization in CTAC improves agreement with reference Agatston risk categories in CSCT compared with manual calcium scoring in CTAC indicating potential for routine CVD risk assessment from CTAC, potentially relieving the need for dedicated CSCT.

## New knowledge gained

Automatic CVD risk categorization based on visual assessment of CAC in CTAC scans shows improved agreement with risk categories determined on dedicated CSCT, as compared to standard manual calcium scoring in CTAC.

## Supplementary Information

Below is the link to the electronic supplementary material.

Supplementary file1 (PPTX 498 kb)

## Abbreviations

CAC: Coronary artery calcification
CTAC: CT attenuation correction
CSCT: Calcium scoring CT
PET: Positron emission tomography
CAD: Coronary artery disease
CVD: Cardiovascular disease
GAN: Generative adversarial network
CNN: Convolutional neural network
