# Supplementary material for: Automated cardiovascular risk categorization through AI-driven coronary calcium quantification in cardiac PET acquired attenuation correction CT
Source: J Nucl Cardiol. 2022 Jul 18;30(3):955–69. doi: 10.1007/s12350-022-03047-9 (PMC10261233; doi:10.1007/s12350-022-03047-9)
Supplement: Supplementary file 1 — Supplementary file1 (PPTX 498 kb) [file 12350_2022_3047_MOESM1_ESM.pptx]

## Slide 1
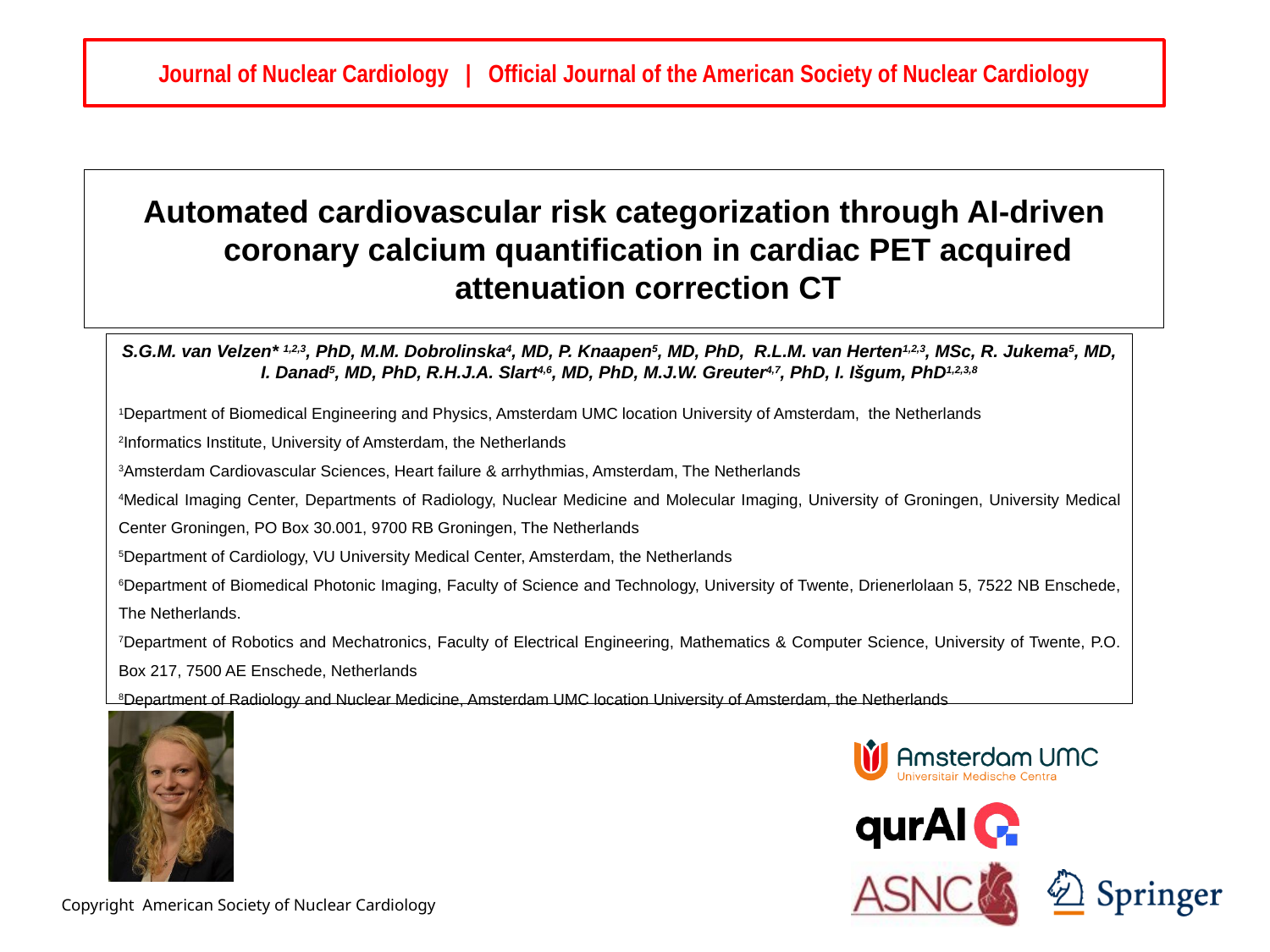

Journal of Nuclear Cardiology | Official Journal of the American Society of Nuclear Cardiology
# Automated cardiovascular risk categorization through AI-driven coronary calcium quantification in cardiac PET acquired attenuation correction CT
S.G.M. van Velzen* 1,2,3, PhD, M.M. Dobrolinska4, MD, P. Knaapen5, MD, PhD,  R.L.M. van Herten1,2,3, MSc, R. Jukema5, MD, I. Danad5, MD, PhD, R.H.J.A. Slart4,6, MD, PhD, M.J.W. Greuter4,7, PhD, I. Išgum, PhD1,2,3,8
1Department of Biomedical Engineering and Physics, Amsterdam UMC location University of Amsterdam,  the Netherlands
2Informatics Institute, University of Amsterdam, the Netherlands
3Amsterdam Cardiovascular Sciences, Heart failure & arrhythmias, Amsterdam, The Netherlands
4Medical Imaging Center, Departments of Radiology, Nuclear Medicine and Molecular Imaging, University of Groningen, University Medical Center Groningen, PO Box 30.001, 9700 RB Groningen, The Netherlands
5Department of Cardiology, VU University Medical Center, Amsterdam, the Netherlands
6Department of Biomedical Photonic Imaging, Faculty of Science and Technology, University of Twente, Drienerlolaan 5, 7522 NB Enschede, The Netherlands.
7Department of Robotics and Mechatronics, Faculty of Electrical Engineering, Mathematics & Computer Science, University of Twente, P.O. Box 217, 7500 AE Enschede, Netherlands
8Department of Radiology and Nuclear Medicine, Amsterdam UMC location University of Amsterdam, the Netherlands
Copyright American Society of Nuclear Cardiology

## Slide 2
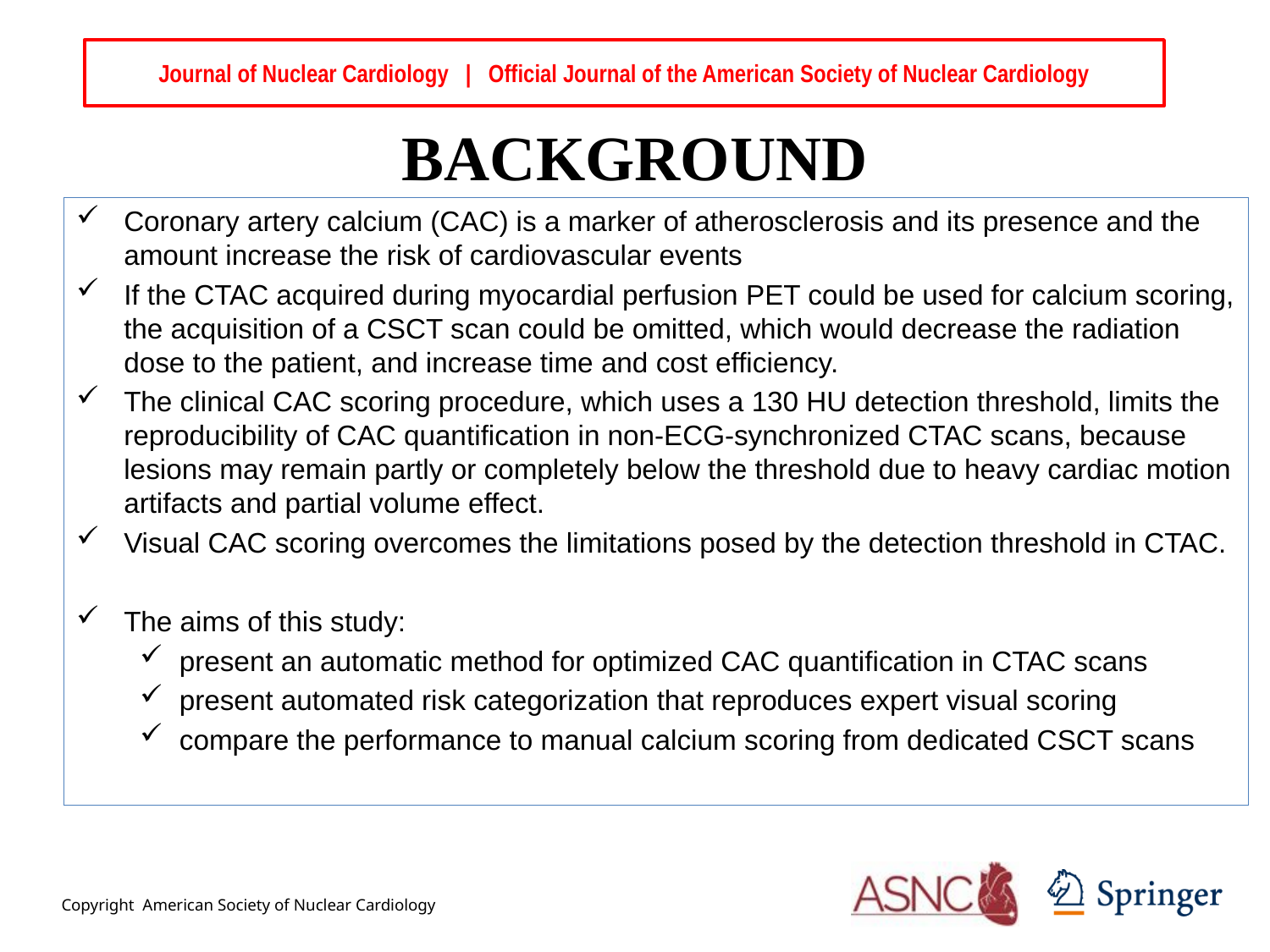

Journal of Nuclear Cardiology | Official Journal of the American Society of Nuclear Cardiology
# BACKGROUND
Coronary artery calcium (CAC) is a marker of atherosclerosis and its presence and the amount increase the risk of cardiovascular events
If the CTAC acquired during myocardial perfusion PET could be used for calcium scoring, the acquisition of a CSCT scan could be omitted, which would decrease the radiation dose to the patient, and increase time and cost efficiency.
The clinical CAC scoring procedure, which uses a 130 HU detection threshold, limits the reproducibility of CAC quantification in non-ECG-synchronized CTAC scans, because lesions may remain partly or completely below the threshold due to heavy cardiac motion artifacts and partial volume effect.
Visual CAC scoring overcomes the limitations posed by the detection threshold in CTAC.
The aims of this study:
present an automatic method for optimized CAC quantification in CTAC scans
present automated risk categorization that reproduces expert visual scoring
compare the performance to manual calcium scoring from dedicated CSCT scans
Copyright American Society of Nuclear Cardiology

## Slide 3
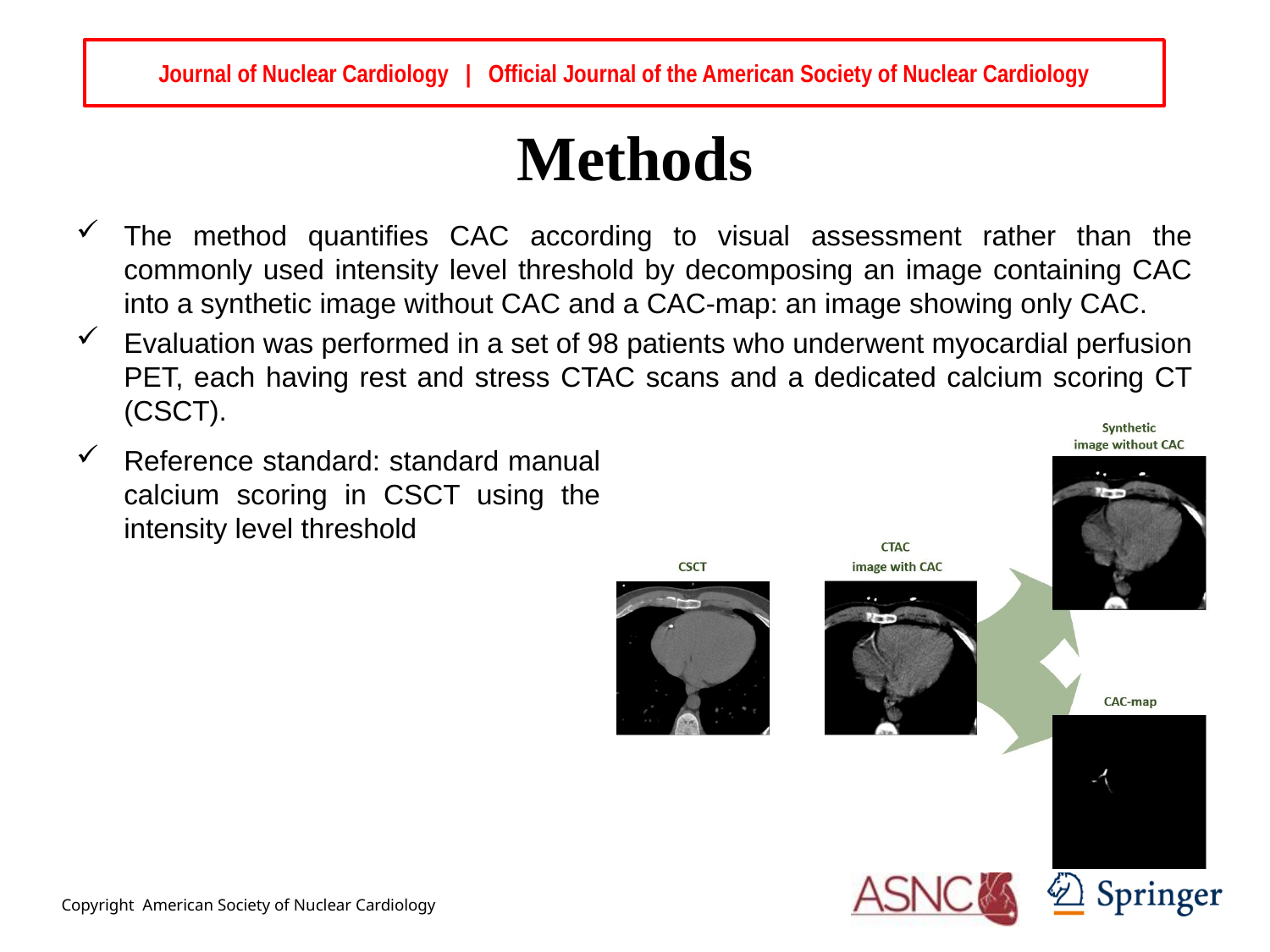

Journal of Nuclear Cardiology | Official Journal of the American Society of Nuclear Cardiology
# Methods
The method quantifies CAC according to visual assessment rather than the commonly used intensity level threshold by decomposing an image containing CAC into a synthetic image without CAC and a CAC-map: an image showing only CAC.
Evaluation was performed in a set of 98 patients who underwent myocardial perfusion PET, each having rest and stress CTAC scans and a dedicated calcium scoring CT (CSCT).
Reference standard: standard manual calcium scoring in CSCT using the intensity level threshold
Copyright American Society of Nuclear Cardiology

## Slide 4
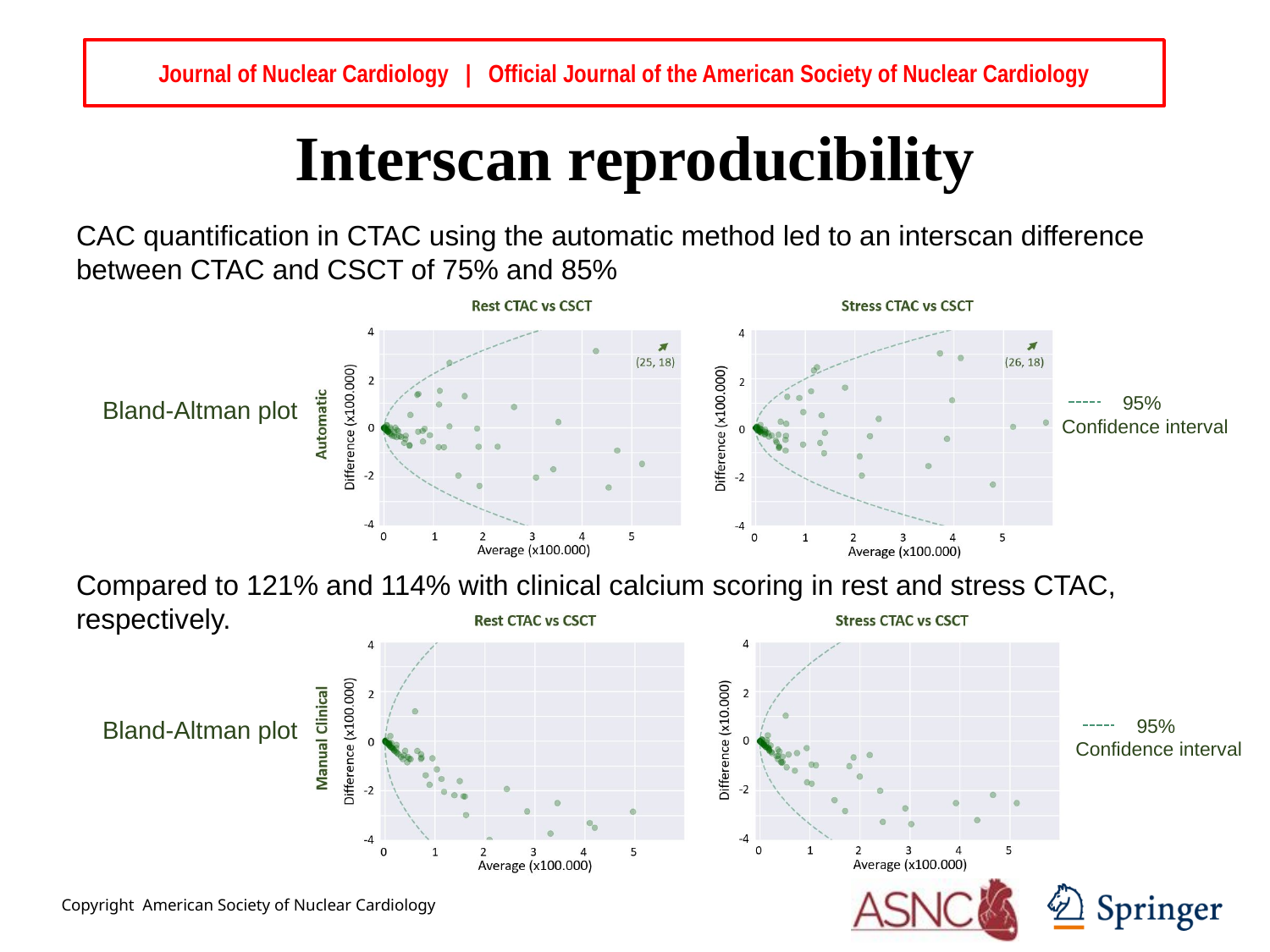

Journal of Nuclear Cardiology | Official Journal of the American Society of Nuclear Cardiology
# Interscan reproducibility
CAC quantification in CTAC using the automatic method led to an interscan difference between CTAC and CSCT of 75% and 85%
Compared to 121% and 114% with clinical calcium scoring in rest and stress CTAC, respectively.
95%
Confidence interval
Bland-Altman plot
95%
Confidence interval
Bland-Altman plot
Copyright American Society of Nuclear Cardiology

## Slide 5
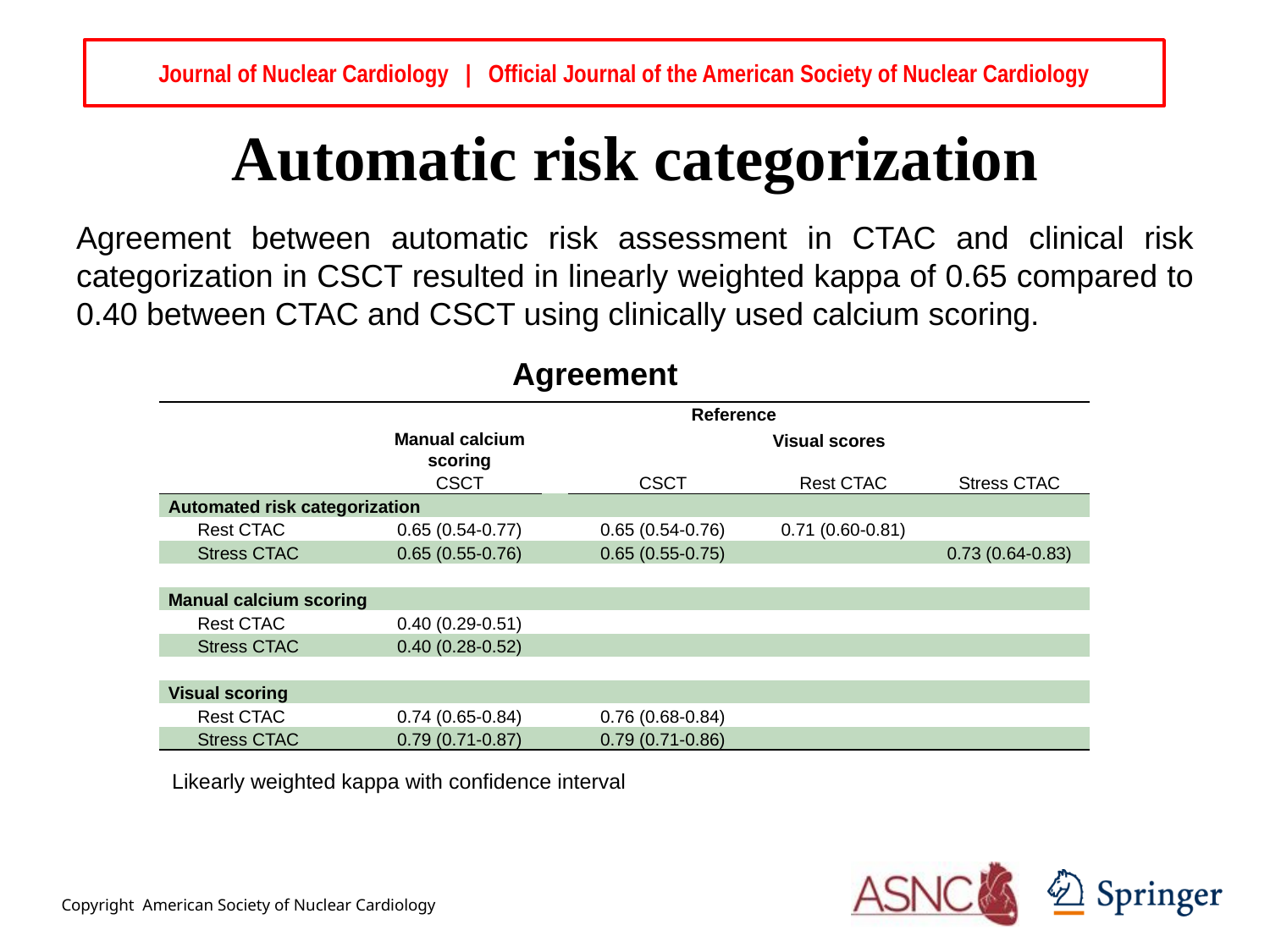

Journal of Nuclear Cardiology | Official Journal of the American Society of Nuclear Cardiology
# Automatic risk categorization
Agreement between automatic risk assessment in CTAC and clinical risk categorization in CSCT resulted in linearly weighted kappa of 0.65 compared to 0.40 between CTAC and CSCT using clinically used calcium scoring.
Agreement
| | | Reference | | | | |
| --- | --- | --- | --- | --- | --- | --- |
| | | Manual calcium scoring | | Visual scores | | |
| | | CSCT | | CSCT | Rest CTAC | Stress CTAC |
| Automated risk categorization | | | | | | |
| | Rest CTAC | 0.65 (0.54-0.77) | | 0.65 (0.54-0.76) | 0.71 (0.60-0.81) | |
| | Stress CTAC | 0.65 (0.55-0.76) | | 0.65 (0.55-0.75) | | 0.73 (0.64-0.83) |
| | | | | | | |
| Manual calcium scoring | | | | | | |
| | Rest CTAC | 0.40 (0.29-0.51) | | | | |
| | Stress CTAC | 0.40 (0.28-0.52) | | | | |
| | | | | | | |
| Visual scoring | | | | | | |
| | Rest CTAC | 0.74 (0.65-0.84) | | 0.76 (0.68-0.84) | | |
| | Stress CTAC | 0.79 (0.71-0.87) | | 0.79 (0.71-0.86) | | |
| | | | | | | |
Likearly weighted kappa with confidence interval
Copyright American Society of Nuclear Cardiology

## Slide 6
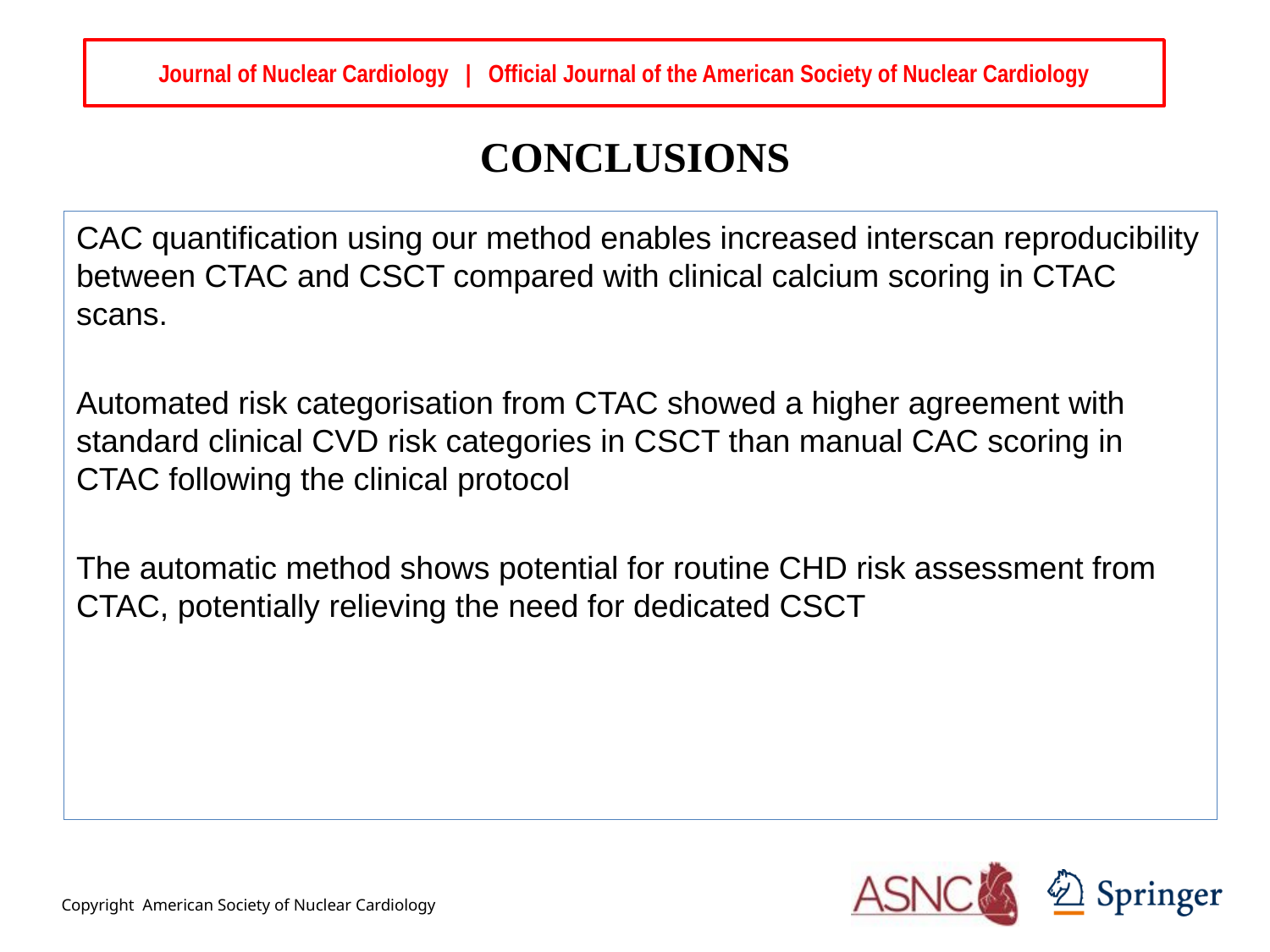

Journal of Nuclear Cardiology | Official Journal of the American Society of Nuclear Cardiology
# CONCLUSIONS
CAC quantification using our method enables increased interscan reproducibility between CTAC and CSCT compared with clinical calcium scoring in CTAC scans.
Automated risk categorisation from CTAC showed a higher agreement with standard clinical CVD risk categories in CSCT than manual CAC scoring in CTAC following the clinical protocol
The automatic method shows potential for routine CHD risk assessment from CTAC, potentially relieving the need for dedicated CSCT
Copyright American Society of Nuclear Cardiology
